# A Case of Von Hippel–Lindau Disease with Bilateral Pheochromocytoma and Ectopic Hypersecretion of Intact Parathyroid Hormone in an Adolescent Girl

**DOI:** 10.1155/2020/8824640

**Published:** 2020-08-07

**Authors:** Rym Belaid, Ibtissem Oueslati, Melika Chihaoui, Meriem Yazidi, Wafa Grira, Fatma Chaker

**Affiliations:** Department of Endocrinology, La Rabta University Hospital, Faculty of Medicine, University of Tunis El Manar, Tunis, Tunisia

## Abstract

Von Hippel–Lindau disease is an autosomal dominant inherited syndrome predisposing to a variety of highly vascularised tumors in different organs. Although bilateral pheochromocytoma was reported in patients with von Hippel–Lindau disease, the coexistence of primary hyperparathyroidism is not a common condition. We report an observation of a primary hyperparathyroidism secondary to an ectopic secretion of intact parathyroid hormone in a 17-year-old girl with von Hippel–Lindau disease and bilateral pheochromocytoma. She presented with a newly diagnosed diabetes mellitus and a severe arterial hypertension. Blood tests disclosed hypercalcemia with increased intact PTH level. Cervical ultrasound and sestamibi scintigraphy were normal. Twenty-four-hour urinary normetanephrine level was highly elevated pointing to a catecholamine-secreting tumor. The abdominal computed tomography showed bilateral adrenal masses. MIBG scintigraphy exhibited a high accumulation of the tracer in both adrenal tumors. Genetic testing revealed a mutation of the VHL gene. The patient underwent a bilateral adrenalectomy. The postoperative outcome was marked by normalization of blood pressure, blood glucose, calcium, and PTH levels. In our case, the elevation of intact PTH and its spontaneous normalization after surgical treatment of pheochromocytomas confirms its ectopic secretion.

## 1. Introduction

Pheochromocytomas are uncommon neuroendocrine tumors that arise from chromaffin cells of the adrenal medulla and produce excessive amounts of catecholamines: epinephrine, norepinephrine, and dopamine [1]. Although most pheochromocytomas are sporadic, more than 25% are associated with an inherited mutation, and this frequency can be as high as 55%, if diagnosed before 18 years of age [2]. In childhood, pheochromocytomas are mostly due to genetic causes including von Hippel–Lindau (VHL) disease, multiple endocrine neoplasia type 2 (MEN2), hereditary pheochromocytoma paraganglioma syndrome, and rarely neurofibromatosis type 1 [3]. Compared to adults, VHL disease is reported to be the most frequent genetic disorder causing pheochromocytomas in children [4]. While bilateral pheochromocytomas were reported in patients with VHL disease, the coexistence of primary hyperparathyroidism is not a common condition [5].

Herein, we report a case of a VHL disease with bilateral pheochromocytoma and an ectopic secretion of intact parathyroid hormone in an adolescent girl.

## 2. Presentation of Case

A 17-year-old girl was referred to our department for the investigation of a newly diagnosed diabetes mellitus and a severe arterial hypertension. She was the first child of consanguineous parents. Her past medical history was unremarkable. Her family history was notable for type 2 diabetes and dyslipidemia but no history of pheochromocytoma, paraganglioma, unexplained sudden death, or condition that may lead to thinking about VHL disease was reported.

As symptoms, the patient complained of headaches, palpitations, diaphoresis, and hot flashes since one month. She reported also asthenia and body weight loss of five kilograms in two weeks.

**Figure 1:**
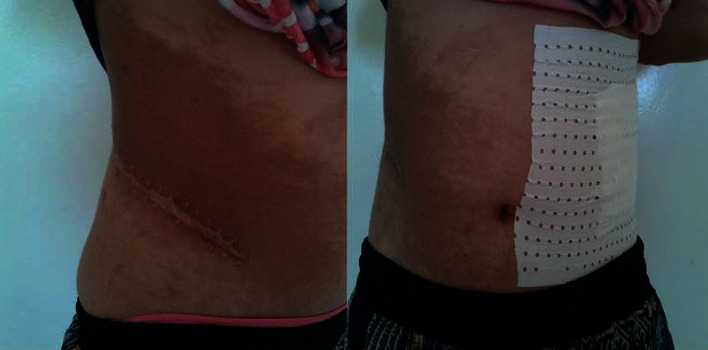
A large abdominal café-au-lait spot.

On examination, she had a body weight of 50 kg, a body height of 154 cm, a body mass index of 21.1 kg/m^2^, a blood pressure of 200/100 mmHg without orthostatic hypotension, a regular pulse of 120 bpm, and a large abdominal café-au-lait spot (Figure 1). Thyroid, abdominal, and neurological examinations were normal.

Twenty-four-hour blood pressure monitoring confirmed the diagnosis of hypertension and the presence of peaks of 230/110 mmHg. Electrocardiogram showed a sinus tachycardia. Capillary glucose level was 2.68 g/l without ketosis.

Fundoscopy showed grade 3 hypertensive retinopathy without any other abnormalities.

The results of biological investigations are shown in Table 1.

The diagnosis of clinically suspected pheochromocytoma was confirmed by the dosage of urinary methoxylated derivatives at 5 times the upper limit of normal. In addition, the diagnosis of diabetes mellitus, dyslipidemia, and primary hyperparathyroidism were made.

**Figure 2:**
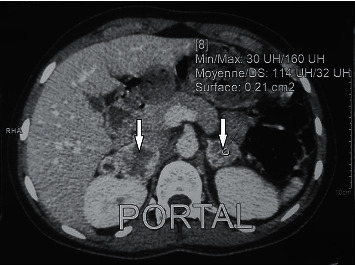
Abdominal computed tomography showing the two adrenal masses (two white arrows).

The abdominal computed tomography (CT) showed two adrenal masses: the first on the right measuring 35 × 55 × 70 mm with a spontaneous density of 30 HU heterogeneously enhanced in the arterial time showing areas of necrosis with an absolute washout of 40%, the second on the left measuring 24 × 27 mm with the same characteristics as the first one (Figure 2). There were neither other localizations nor lymph nodes. Metaiodobenzylguanidine (MIBG) scintigraphy exhibited a high accumulation of the tracer in both adrenal tumors with no other localization. Cervical ultrasound and 99mTc-sestamibi scintigraphy were normal. Cardiac ultrasound was normal.

The diagnosis of multiple endocrine neoplasia type 2a was highly suspected, and the patient underwent a molecular investigation. DNA analysis did not find a RET proto-oncogene mutation. However, it showed a missense mutation c.191G > C (p.Arg64Pro) in exon 1 of the VHL gene on chromosome 3. The diagnosis of bilateral pheochromocytoma in the setting of VHL disease was established. Abdominal CT scan and craniospinal magnetic resonance imaging with contrast did not show any cysts or other tumors.

The patient was treated with an alpha blocker, prasozine, a calcium channel blocker, a beta blocker, and insulin at a daily dose of 1.4 units/kg. She underwent a bilateral adrenalectomy in two steps. The pathological examination confirmed bilateral adrenal pheochromocytomas. Replacement therapy with hydrocortisone was initiated after surgery.

The postoperative outcome was determined by the spontaneous normalization of blood pressure, blood glucose, calcium, and PTH levels. The patient remained asymptomatic with no evidence of local recurrence or distant metastasis during the 12 months of follow-up. The family screening for VHL has not been performed yet.

## 3. Discussion

VHL disease is a dominantly inherited familial cancer syndrome caused by a germline mutation in the VHL tumor suppressor gene and predisposing to a variety of benign and malignant neoplasms most frequently retinal, central nervous system and spinal hemangioblastomas, renal cell carcinoma (RCC), pheochromocytoma, and pancreatic tumors [6, 7]. While central nervous system and retinal hemangioblastomas are the earliest expressions of the VHL syndrome, pheochromocytoma may be the first manifestation of the disease especially in children and adolescents as it was the case of our patient [6–8]. The frequency of pheochromocytoma in VHL syndrome is about 10 to 20% [6].

Families with VHL disease have been divided into two subtypes: VHL type 1 and VHL type 2, based on the likelihood of developing a pheochromocytoma. The presence of pheochromocytoma defines types 2 VHL disease. This latter is subdivided based on the risk of developing RCC. Type 2A and 2B families have a low and high incidence of RCC, respectively, whereas VHL type 2C kinds are characterized by the development of pheochromocytoma without any other manifestations of the disease [7]. However, late onset of other attacks is possible, and a follow-up, even spaced, is required [9].

Genotype-phenotype correlations have been documented for this disorder, and specific mutations are associated with the appearance of tumors in certain organs. While most type 2 families were reported to be more likely carrying a missense mutation in the VHL gene, most type 1 families are affected by truncating or deletion mutations [10]. In our case, the presence of pheochromocytomas and the missense mutation in VHL gene suggested type 2 VHL disease. Moreover, the mutation found in our patient, p.Arg64Pro, has been described in patients with isolated pheochromocytoma, associated with RCC or with pancreatic neuroendocrine tumor [11–13]. However, our patient did not present any sign of RCC or pancreatic neuroendocrine tumor during the 12 months of follow-up. This evaluation may be early in our case as RCC and pancreatic neuroendocrine tumors in patients with VHL disease generally appear at more advanced ages (around 25–35 years) [7].

Pheochromocytoma in VHL disease tends to be seen at a younger age and is more frequently multifocal, as in our patient and may be extra-adrenal [14, 15]. In most published cases, the mean age at presentation was about 30 years, but very young cases have been described, the youngest before 5 years [7, 10]. In addition, VHL-associated pheochromocytomas are less likely to be associated with symptoms or biochemical evidence of catecholamines production compared with those occurring in patients without VHL [16, 17]. In a report of the National Institute of Health about 64 patients with VHL disease and pheochromocytomas, a total of 106 tumors were identified. Of these, 12% originated outside the adrenal gland, and 35% of the patients were asymptomatic, without hypertension or evidence of increased catecholamines production [15]. This was not the case of our patient who had sustained severe hypertension associated with the classic symptoms of pheochromocytoma (palpitation, sweating, and hot flashes), hypokalemia, and a secondary diabetes mellitus. In our case, as reported in the literature [16, 17], a remarkable remission of diabetes mellitus and an improvement of lipid profile were noticed after tumor removal confirming the secondary character of these two metabolic disorders. In fact, catecholamine excess affects insulin secretion, decreased glucose uptake in the peripheral tissues, and increased insulin resistance leading to impaired fasting glucose or overt diabetes mellitus [18]. Moreover, the increase in catecholamine production may be responsible for decreased inhibition of lipolysis by insulin and decreased activity of lecithin-cholesterol acyltransferase, an enzyme which breaks down free cholesterol [1].

The risk of malignancy is low. Less than 5% of all pheochromocytomas in VHL disease are malignant [7]. In our case, neither distant metastasis nor lymph nodes were found, but a long term follow-up should be carried out.

Measurement of plasma or urinary metanephrines and normetanephrines is the gold standard in diagnosing pheochromocytoma and can also provide important diagnostic information [1]. In fact, in a study carried out by Eisenhofer et al. [14], comparing the clinical and biochemical characteristics of pheochromocytomas in multiple endocrine neoplasia type 2 versus the VHL syndrome and including 19 and 30 patients with these disorders, respectively, VHL patients almost exclusively produced normetanephrines. So, a high normetanephrines-to-metanephrines ratio is expected in patients with VHL disease, as was found in our patient. Furthermore, in comparison with MEN2 tumors, VHL tumors had lower total tissue contents of catecholamines and expression of tyrosine hydroxylase (TH), the rate-limiting enzyme in catecholamine synthesis. They also had much lower expression of phenylethanolamine N-methyltransferase (PNMT, the enzyme that converts norepinephrine to epinephrine) and tissue stores of epinephrine [14]. Regarding the histopathological features, VHL-associated pheochromocytomas are characterized by a thick vascular tumor capsule and are in contrast to MEN 2 not associated with adrenal medullar hyperplasia [19].

While bilateral pheochromocytomas were reported in patients with VHL disease, the coexistence of primary hyperparathyroidism as in our case is not a common condition [5]. Hypercalcemia associated with pheochromocytoma has been documented and is thought to be caused by several mechanisms. First, elevated catecholamines can activate the PTH receptor resulting in catecholamine-induced osteoclastic bone resorption, but in contrast to our case, the PTH level is not elevated [20]. Secondly, hypercalcemia can be due to the production of PTH-related peptide (PTH-rp) by the tumor which was doubtless not the case in our patient as the level of intact PTH was elevated [21]. Furthermore, intact PTH in our case was quantified using a 3rd generation chemiluminescence immunoassay which does not recognize PTH-rp.

Third, parathyroid adenoma can be a part of multiple endocrine neoplasia that was, however, rarely reported in VHL disease [5]. The negativity of the topographic investigations and the spontaneous normalization of intact PTH after surgical treatment are against this hypothesis. Finally, although the exact physiopathological mechanism is not clear, the fact that both serum calcium and PTH levels were elevated before surgery and became normal after the removal of the pheochromocytomas strongly suggests that the tumor itself was secreting PTH or a substance that stimulates excessive PTH secretion by the parathyroid glands. Only few cases of ectopic hormonal secretion by pheochromocytoma such as adrenocorticotropic hormone (ACTH), calcitonin, parathyroid hormone (PTH), vasoactive intestinal peptide (VIP), and growth hormone-releasing hormone (GHRH) were reported [22, 23]. Unfortunately, immunohistochemistry assay for PTH in the tumor tissue was not available to clarify this question in our case.

## 4. Conclusion

This report highlights the rare case of ectopic intact PTH secretion by a bilateral pheochromocytoma in an adolescent girl with VHL disease. We consider that controlling calcium and PTH after adrenalectomy is useful if the topographic assessment of primary hyperparathyroidism is negative.

To the best of our knowledge, our patient is the second youngest reported childhood VHL case in the literature, presenting with a bilateral pheochromocytoma secreting ectopic intact PTH. Genetic testing and a meticulous follow-up are necessary for the diagnosis of the associated comorbidities in VHL disease.

## Figures and Tables

**Table 1:** Biological parameters before and after surgery.

	Before surgery	After surgery	Reference ranges
Fasting glucose level (mmol/l)	16.39	4.78	4.12–5.55
Glycated hemoglobin (%)	10.12	5.91	≤5.6
Total cholesterol (mmol/l)	11.83	5.23	3.1–5.18
Triglycerides (mmol/l)	2.43	1.21	0.57–1.71
Natremia (mmol/l)	135	140	135–145
Kalemia (mmol/l)	3.3	4	3.6–5
Creatinine (mg/L)	6	6	4–13
Calcium (mg/L)	111.2	98	88–105
Phosphate (mg/L)	44	36	27–45
Albumin (g/l)	47.22	—	35–52
Intact PTH^*∗*^ (*μ*g/L)	182	54	11–62
TSH (mUI/L)	0.625	—	0.12–3.4
FT4 (ng/dl)	1.31	—	0.71–1.85
Calcitonin (ng/L)	2.70	—	<10.00
24 h-urine NMN (*μ*g/24)	3150	570	30–600
24 h-urine MN (*μ*g/24)	40.2	12	20–345
24 h-urine sodium (mmol/24 h)	88.5	—	
24 h-urine potassium (mmol/24 h)	60	—	
24 h-urine calcium (mg/24 h)	200	—	
24 h-proteinuria (g/24)	0.3	—	<0.3

PTH: parathormone, TSH: thyroid stimulating hormone, FT4: free-T4, NMN: normetanephrines, MN: metanephrines. ^*∗*^The intact PTH dosage was made using 3^rd^ generation chemiluminescence immunoassay.

## Data Availability

The data used to support the findings of this study are available from the corresponding author upon request.
